# “Learning from the experts” – a novel advanced cadaveric course for Gynaecological Oncology (GO) Cytoreductive Surgery

**DOI:** 10.52054/FVVO.14.3.036

**Published:** 2022-09-30

**Authors:** M Sideris, A.M. Elshaer, R.L. Johnson, S Kotwal, S Mehta, A Quyn, R Saunders, J Tiernan, V Upasani, G Theophilou

**Affiliations:** St. James University Hospital, The Leeds Teaching Hospitals, NHS, UK

**Keywords:** Simulation-based learning (SBL), cadaveric course, cytoreductive surgery, ovarian cancer, postgraduate education

## Abstract

**Background:**

Ovarian cancer cytoreductive surgery necessitates the use of advanced Simulation-Based Learning (SBL) to optimise skill-based teaching and achieve technical proficiency.

**Objective:**

We describe and appraise the role of a novel postgraduate cadaveric course for cytoreductive surgery for advanced ovarian/fallopian tube or primary peritoneal cancer.

**Materials and Methods:**

Several consultant-level surgeons with expertise in upper gastrointestinal, colorectal, hepatobiliary and urological surgery, were invited to teach their counterpart gynaecological oncology (GO) surgeons. The 2-day course curriculum involved advanced dissections on thiel-embalmed cadavers. All dissections included applicable steps required during GO cytoreductive surgeries.

**Outcome measures:**

We used a feedback questionnaire and structured interviews to capture trainers and delegates views respectively.

**Results:**

All delegates reported a positive educational experience and improvement of knowledge in all course components. There was no difference in the perception of feedback across junior versus senior consultants. Trainers perceived this opportunity as a “2-way learning” whether they got to explore in depth the GO perspective in how and which of their skills are applicable during cytoreductive surgery.

**Conclusions:**

Collaborating with other surgical specialities promotes a “learning from the experts” concept and has potential to meet the rapidly increased demand for multi-viscera surgical excellence in GO surgery.

**What's new?:**

The concept of involving experts from other surgical disciplines in advanced cadaveric courses for cytoreductive surgery in ovarian cancer, will solidify the effort to achieve excellence in the GO training. Such courses can be essential educational adjunct for most GO fellowships.

## Manuscript

Since the establishment of the Gynaecological Oncology (GO) surgery as a subspecialty 50 years ago (Hoffman and Bodurka, 2009), significant advances in the field have mandated the need for surgeons to acquire a melange of complex surgical skills in order to practice radical surgery safely. This includes advanced anatomy knowledge, as well as familiarity and exposure to techniques and surgical approaches that traditionally have been practiced by other surgical specialties. A classic example is ovarian cancer cytoreductive surgery, where keeping surgical morbidity and mortality to as minimal as possible, whilst achieving complete cytoreduction, requires the GO surgeon to be familiar and competent with principles of advanced lower gastrointestinal (GI) colorectal, urological, upper GI and or hepatobiliary (HPB) surgery. Meanwhile, surgical equipment and techniques advance which subsequently generates higher expectations from patients and public. Hence, it is becoming vital for the future generation of GO surgeons not only to be technically accomplished, but more importantly, to understand their limitations and have a clear mindset where and how to seek expert help if needed. This ultimately aims to reduce surgical morbidity whilst achieving complete cytoreduction.

Current GO subspecialty curricula in the UK and several EU countries entail formal exposure in urology and colorectal surgery (Cibula and Kesic, 2009). However, GO trainees frequently acquire or master such specialty-related (GI/urological) skills whilst operating with only GO surgeons. In several cytoreductive surgeries GO surgeons invite advance specialists from other surgical fields to assist with complex elective operating, to achieve complete cytoreduction. Despite this, there is still a notable variation in terms of what GI/ HPB or urological procedures each GO consultant is familiar to perform, and to what extent of complexity. Those differences are observed not only between different units across the UK, but also between GO surgeons within the same unit.

The increased demand for complete cytoreductive in GO surgery necessitates the introduction of advanced postgraduate simulation-based learning (SBL) to optimise skills-based teaching and allow trainees to safely achieve technical proficiency. The multidisciplinary nature of ovarian cancer cytoreductive surgeries, dictates a reassessment of the needs assessment process whilst designing SBL postgraduate courses. “Learning from the experts” defines a novel approach in GO SBL postgraduate course design, where surgeons from several specialities teach GO surgeons how to perform procedures that belong to their area of expertise. This aims to achieve excellence in learning outcomes, and to enhance GO collaboration and communication with other surgical specialities. In this regard, we present our experience from one of the first reported advanced cytoreductive cadaveric courses.

## Methods

### Trainers/Trainees

Eight consultant-level surgeons with expertise in upper GI, colorectal, HPB and urological surgery, were invited to teach GO surgeons from several tertiary oncology centres in the UK. We invited trainers who have been regularly involved in advanced GO cytoreductive surgery for at least 3 years; this was to ensure that there was adequate understanding of the skillset applicable in GO. There were 2 trainers from each surgical speciality. All trainers came from a single tertiary oncology centre to ensure there are no gross variations in their own (specialty-related) practice.

For this pilot course, delegates were exclusively consultant level GO surgeons with <5, 5-10 or >5 years of experience in cytoreductive surgery. Delegates came from different units across the UK to achieve a good representation of different local approaches in cytoreductive surgery. This refers to the extent of upper GI/ colorectal/ HPB/ urological procedures that GO Surgeons are performing independently in their routine practice.

### Set up/curriculum

**Figure 1:**
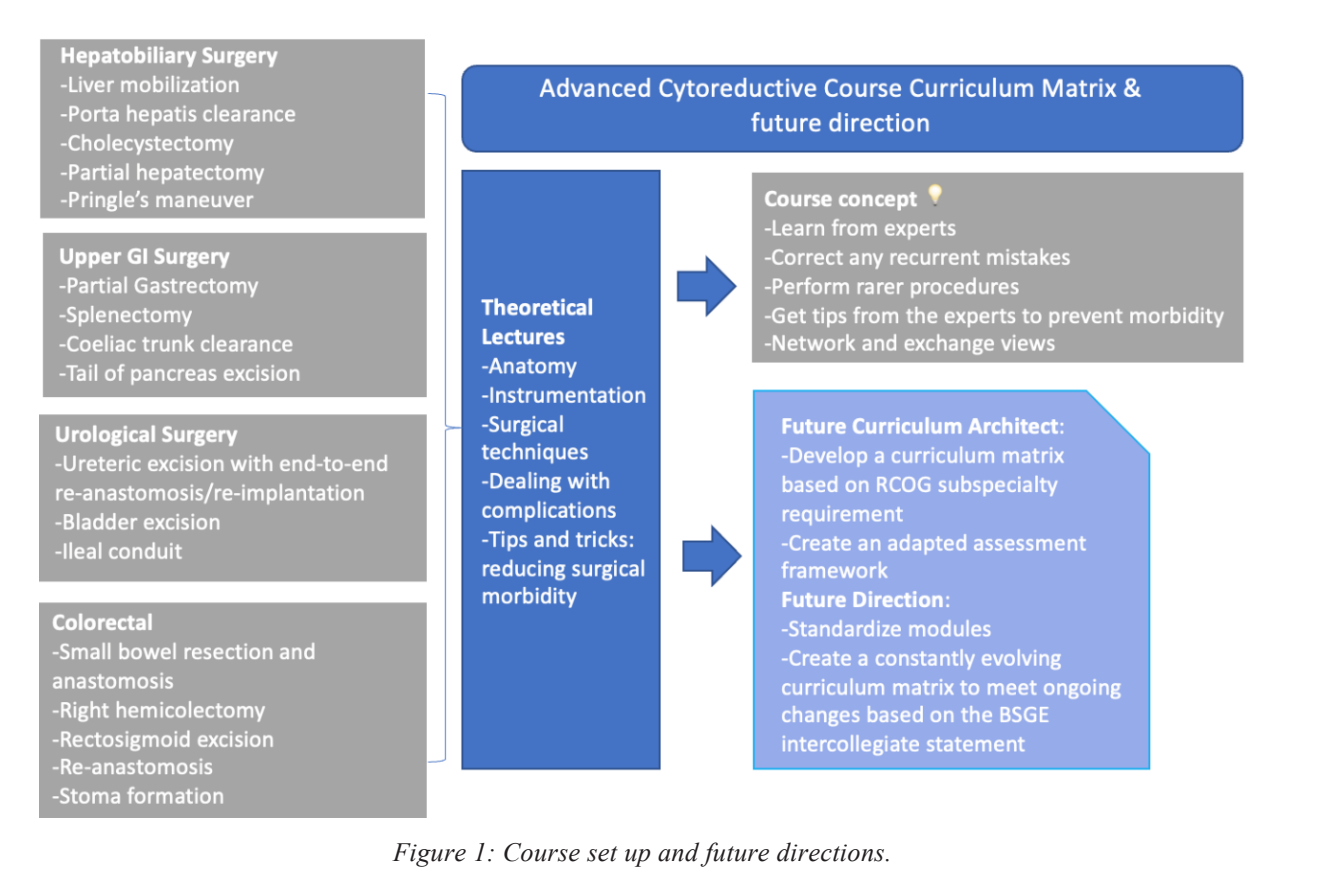
Course set up and future directions.

The curriculum was spread across the two days and divided in four axes covering all four subspecialities (two per day). For each of the subspecialty axis, there was an introductory interactive lecture focused on the relevant applied anatomy and the principles for safe dissection, as well as common pitfalls that can arise during those surgical approaches. In each lecture there was a featured section on when the GO surgeon should ask expert input from other specialities (Figure 1).

### Venue/modality of SBL

We used 2 thiel-embalmed cadavers. All dissections took place in the cadaveric laboratory at the University of Leeds, according to local ethical practice and standard operational procedures. Each trainee performed the full range of procedures listed in Figure 1. For each cadaver there were 2 trainers at the time.

Feedback from the delegates: we designed a six-domain detailed questionnaire which covered every single aspect of the course (Appendix 1). Domain one included 21 questions which explored the views of each GO consultant towards the course and to determine the course’s ability to advance essential skills in cytoreductive surgery. It also included questions such as anonymised demographics and previous years of experience. Domain two-five included four questions on module specific feedback aiming to explore which aspect/s of the 4 axes should be expanded in the course curriculum. Lastly, domain six included five open-ended questions such as areas to improve or any further feedback. All questions used a Likert scale 0-5.

Feedback from trainers: we conducted an informal structured interview with all the trainers from each subspecialty (Table I). The overall direction of interview questions was based on three axes: oncological outcome, working as a team and patient safety.

**Table I:** Thematic axes for delegates’ and trainers’ feedback.

Thematic Axes	Delegates Views
Axis 1	Overall perception of the GO surgeons’ baseline (prior to the course) skills/knowledge applicable to each subspecialty
Axis 2	Overall perception of improvement in subspeciality related skills (Upper HPB, GI, Urology and Colorectal Surgery)
Axis 3	Overall perception of the role of theoretical lectures towards optimising learning during the course
Axis 4	Any observed differences in the perception towards the course across different seniority stages of GO Surgeons and applicability of the course modules for a junior or senior consultant
Axis 5	Any observed differences in the perception towards the course across different region of practice of each GO surgeon
Trainers’ structured interview thematic axes	
Axis 1	Oncological outcomes: whether such courses improve GO surgeons’ skills related to their own (trainers) field
Axis 2	Working as a team: whether such courses improve communication between different specialities and create an op- portunity for mutual understanding of the aim of cytoreductive surgery for optimal results
Axis 3	Patient safety: whether this course help GO Surgeons to realise their potential limitations in each subspeciality and know better when to seek for help pre-operatively or even intra operatively

### Data synthesis (delegates’ views)

We used a mixed quantitative and qualitative approach to summarise delegates’ feedback. Initially we calculated median scores using Microsoft Excel (quantitative analysis). Following this, we shortlisted the most important (according to the authors) five thematic axes (qualitative analysis) (Table I).

Based on the median score (Likert scale 0-5), for each thematic axis we summarised the results with the terms “Minimal” (0-1), “Average” (2-3), or “Good” (4-5).

## Results

Seven delegates attended the course and provided feedback. Appendix 1 represents the overall feedback received from the delegates; Table II represents the thematic analysis summary of the delegates’ feedback. Table III represents the thematic analysis summary of the trainers’ feedback around oncological outcomes, patients’ safety and working as a team.

**Table II:** Delegates views (0-1=minimal, 2-3=average, 4-5=good).

Thematic Axis	HPB	Upper GI	Urology	Colorectal Surgery
Overall perception of the GO surgeons’ baseline (prior to the course) skills/knowledge applicable to each subspecialty	Average	Average	Average	Good
Overall perception of improvement in subspeciality related skills (Upper HPB, GI, Urology and Colorectal Surgery)	Good	Good	Good	Good
Overall perception of the role of theoretical lectures towards optimising learning during the course	Good	Good	Good	Good
Any observed differences in the perception towards the course across different seniority stages of GO Surgeons and applicability of the course modules for a junior or senior consultant	Nil	Nil	Nil	Nil
Any observed differences in the perception towards the course across different region of practice of each GO surgeon	Nil	Nil	Nil	Nil

**Table III:** Trainers’ views on the course (0-1=minimal, 2-3=average, 4-5=good).

Thematic Axis	HPB	Upper GI	Urology	Colorectal Surgery
[oncological outcomes] whether such course improve GO surgeons’ skills related to their own (trainers) field	Good	Good	Good	Good
[working as a team] whether such courses improve communication between different specialities and create an opportunity for mutual understanding of the aim of cytoreductive surgery for optimal results	Good	Good	Good	Good
-[patient safety] whether this course help GO Surgeons to realise their potential limitations in each subspeciality and know better when to seek for help pre-operatively or even intra operatively	Average	Average	Average	Good

## Discussion

### GO postgraduate training: requirements and aims

Gynaecological Oncology has noted remarkable advances in the last few decades, and this has raised trainees’ expectations (Hoffman and Bodurka, 2009; Cibula and Kesic, 2009). Despite significant variations in the postgraduate training curricula across the US, EU or UK, the global principles are similar. Hoffman and Bodurka (2009) articulates the American perspective of GO education around three pillars; firstly, the provision of technical skills which predominantly comprise of advanced surgical skills along with relevant expertise to support the patients pre- and post- operatively. The second pillar emphasises on the importance of non-technical skills (NTS). NTS in GO include the application of clinicians’ interpersonal skills to build an entrusted relationship with their patients; it also includes their ability to coordinate several ancillary services and provide comprehensive care as part of a multidisciplinary team. The third pillar comprises of the infrastructure that is present to support the required services, and this is mainly related to financial support towards equipment and human resources to achieve excellence in surgical training and care provision.

Cibula and Kesic (2009) describe the European viewpoint on GO postgraduate training based on three main components: trainers, trainees, and training centres. This review article underlines the need of global standardisation of GO training and assessment, underpinning the importance of the use of various SBL modalities (educational tools), as well as educational resources (videos, online lectures etc). This would serve as a training facilitator and skill accelerator, especially in a unit with low GO surgical volume and thus less frequent GO surgical exposure. In a similar context with Hoffmann and Bodurka (2009), in this review summary, there is increasing emphasis on NTS and their formal assessment. A classic example is the use of non-technical skills in surgical training (NOTTS) tool, which allows valid and reliable observation and assessment of trainees’ decision making, communication, teamwork, situation awareness and leadership. Finally, Cibula and Kesic (2009) explain the importance for trainers to be adequately qualified to train their juniors, and this should be strictly defined against a formal framework.

However, in both schools -American and European- it seems that there is slim emphasis on multi-specialty involvement to optimise surgical cytoreductive outcomes. By “multi-specialty” we refer to the direct involvement of experts from other surgical specialities who assist, and train GO surgeons to perform procedures that are not in the GO area of expertise. The British Gynaecological Cancer Society (BGCS) along with the Royal College of Obstetricians and Gynaecologists (RCOG) define the GO sub-specialty curriculum syllabus (RCOG, 2019). This syllabus clearly incorporates placements of GO trainees in several other surgical specialities such as colorectal or urology along with the technical skills-based competencies that the GO trainee should acquire by the end of the subspecialty program. However, despite this, there is still a notable variation across several GO tertiary centres across the UK or occasionally within the same centre, as to which of those procedures are performed independently by GO surgeons without further subspecialist input and which are performed by, or in collaboration, with a specialist surgeon outside of GO.

In an effort to standardise multidisciplinary cytoreductive surgery for advanced ovarian, fallopian tube or primary peritoneal cancer, the British Society for Gynaecology Endoscopy (BSGE) in conjunction with several surgical colleges, published a recent (intercollegiate) statement to suggest governance models to support patients’ safety in cytoreductive surgeries(Maxwell- Armstrong et al., 2021). This statement is the first formal approach to set a framework for joint working between GO and colorectal or upper GI surgeons. This statement introduces the idea of working closely with colorectal surgeons who have a relevant GO specialty interest and subsequently their job plan includes dedicated sessions for cytoreductive surgery. It also introduces the idea of joint MDTs to improve pre-operative planning i.e. improve prediction of when the presence of specialist surgeons outside of GO are required. Furthermore, it introduces the idea of a joint approach towards enhanced recovery practice (ERAS) which expands to a concept of multi- specialty involvement to achieve continuity of care in the post-operative period. This includes joint morbidity reporting which allows to acquire clear comparative data in morbidity and mortality.

### Simulation-Based Learning (SBL) modalities: the role of cadaveric dissections in GO

SBL modalities in undergraduate and postgraduate education vary significantly (Theodoulou et al., 2018) depending predominantly on the primary learning outcomes (Prideaux, 2003; Sideris et al., 2021). Cadaveric dissection in advanced GO education (anatomy) and training (Selcuk et al., 2019) have been well established globally. In the UK, thiel-embalmed cadavers (TEC) have been widely used for several purposes. TEC have been associated with a series of advantages which offer superior simulation experience and an expanded calibre of applications. Highly maintained tissue fascial planes, colour plasticity and elasticity (Mitsugashira et al., 2022) improve fidelity of simulation for several surgical specialities. TEC offer a wide range of adaptations to their preservations’ standard protocol which allows expansion of their application from traditional surgical dissections to fibre-optically guided intubation (Laszlo et al., 2018) and radiofrequency ablation techniques (Liao and Wang, 2019). Researchers in the University of Dundee have been pioneering the use of TEC in testing innovative devices with great experience.

Enthusiasts like Barton et al. (2009) introduced the first formal cadaveric anatomy course tailored for GO trainees in the UK in 2008 (Barton et al., 2009). Based on their experience, the anatomy knowledge of GO subspecialty trainees was weak prior to that workshop but improved significantly as a result of intense dissections. An interesting comment from that study, is the authors preference towards “soft preserved cadavers” than formalin fixed, which seems to be standard practice in most cadaveric laboratories in the UK. Although evaluation and assessment from the course delegates indicates good short-term improvement of their anatomy knowledge, there is a long-standing argument whether these courses contribute to long-term acquisition and retention of anatomy knowledge. Gordinier et al. (1995), almost 3 decades ago, supported the use of cadavers as a formal educational tool which objectively and subjectively contributes to increase the knowledge of pelvic anatomy of GO residents as part of the formal USA training fellowship program. Another classic example of the role of cadaveric courses in GO comes from the Flemish Society of Obstetrics and Gynaecology. Tjalma et al. (2013) describe their positive experience in delivering a cadaveric laparoscopic course for gynaecology residents, aiming to advance their anatomy knowledge and surgical skills; most delegates reported a positive experience.

### Cadaveric courses: what is the impact in clinical practice?

The role of cadaveric courses in modern GO education becomes increasingly vital, considering several medical schools tend to limit the exposure of their students to dissections, and even more in cadaveric dissections. This was an argument posed many years ago (Snelling et al., 2003), when medical students reported concerns regarding stress generated by cadaveric dissections. However, this has not been the case with postgraduate trainees. Given the limited exposure in medical school to anatomy teaching, along with the increasing expectations and radicality of modern GO, such courses are deemed essential to maintain safety and high standards in surgical education.

Another important point that underlines the importance of such courses is the fact that trainees in obstetrics and gynaecology tend to have limited exposure in gynaecological procedures throughout their training, a problem which has been magnified by the COVID 19 pandemic (Mallick et al., 2021). Subsequently their surgical experience in advanced pelvic dissection when they enter the GO subspecialty program is limited often necessitating more time spend in GO training programmes in order to reach surgical competence in GO (Barton et al., 2009). Therefore, cadaveric simulation is essential to ensure GO surgeons are adequately exposed in Several procedures and build confidence prior to the operating theatre. This is also vital, as it can shorten their learning curve and expedite learning.

Multidisciplinary cadaveric courses are vital for an additional reason. As discussed later, such initiatives can act as a “bridge” between clinicians of different surgical disciplines and build a solid working relationship, where each team (i.e., colorectal, urology etc.) understand the needs of our sub-speciality. And this is the direction BGCS is heading at present with a growing demand of “experts” involvement in GO advanced cytoreductive surgery. An important point to raise though, is even though SBL has an established role, improving directly surgical skills and facilitating learning curve, it is difficult to quantify its (SBL) impact on patients’ outcomes directly. Ultimately this should be the outcome of each SBL related study, however only a few research set ups are designed to answer this question. A systematic review (Zendejas et al., 2013) published in 2013 showed a direct benefit of patients when healthcare providers were instructed by SBL, however this study acknowledged a series of limitations including reported heterogeneity of the included studies.

### Delegates views on the role of our course

Overall, everyone reported a positive educational experience and improvement of knowledge in all the components of the course. There was no difference in the perception of feedback across junior versus senior consultants, and everyone expressed an interest for the course to be adapted and implemented in the UK GO subspecialty curriculum. Interestingly, delegates reported an even improvement of their knowledge in HPB, upper GI, colorectal and urology. This suggests a uniform level of HPB/upper GI, colorectal and urology expertise within the GO cohort and poses an argument as to which of the above areas GO surgeons are more familiar with. However, a small cohort of 7 delegates did not allow further exploration of the sample. Similarly, we could not assess any differences across different areas of practice in the UK due to sample limitations. Delegates found introductory lectures useful, and their recommendation is to design a stricter pinpoint matrix of procedures for each subspecialty, that are mostly related to GO cytoreductive surgery.

### Trainers (multi-disciplinary) views on the course. Learning from the experts: taking a step further in GO postgraduate education

Trainers expressed interesting views; they perceived this opportunity as a “2-way learning” whether they got to explore in depth the GO perspective in how and which of their skills are applicable during cytoreductive surgery. It also challenges experts from different surgical specialities to reflect on how they can adapt their skills and approach to optimise successful cytoreductive surgery. This is the first reported course to create a “two-way” learning environment and raise expectations towards designing and implementing guidelines of standardised cytoreductive practice across the 4 subspecialities which are specifically addressed for GO related procedures. The Royal College of Surgeons (RCS) can take an important role to lend significant expertise in surgical practice via their trainers, which could help train GO specialists to optimising their skills in areas that are not the “bread and butter” of their everyday practice. And by “optimise”, we refer to both the improvement of our own GO independent skills, but also, the understanding of our limitations as to when, where and how to ask for help and plan surgery safely from the earliest possible stage.

Our course can be developed in line with the recent intercollegiate statement towards a novel skills-based curriculum that adopts the vision of providing multidisciplinary expert- based education, focused on achieving optimal surgical results. It reflects a novel approach in GO education and recognises the current trend towards advancing practice in cytoreductive surgery. Expert trainers clearly enjoyed this opportunity, and more importantly they expressed a clear interest to formalise education not only for GO specialists, but also to involve and educate their trainees in GO cytoreductive surgery at the earliest possible stage.

### Future directions & development (a new era)

COVID 19 has implemented new rules and expectations in medical and surgical education which have been supported by several innovations (Dedeilia et al., 2020; Sideris et al., 2020). Face-to- face SBL education has been eliminated to cover only the essential needs, however, undoubtedly advanced postgraduate courses are still taking place as before. Hence, such courses will continue to evolve rapidly especially with the view to cover compromised surgical training opportunities. Implementation of several novel adjuncts like online lectures and videos as part of cadaveric courses will be crucial, to both eliminate contact as well as optimise learning during the course and maximise its educational benefit. Our current view is that for such advanced postgraduate courses, cadaveric dissections, will continue to be necessary and could not be imminently replaced. Hence a face-to-face live demonstration approach will be still the case, however novel technologies implemented during covid times can facilitate learning, or perhaps limit the time exposure that traditionally was required in live cadaveric demonstration. High fidelity simulation will still have a place in advanced postgraduate education, until proven otherwise.

This course ran on a pilot mode, and indeed there is a long way to go before it is standardised in the core curriculum. However, its foundation needs assessment is robust, and it has a unique potential to evolve in an essential GO curriculum item. Reshaping its curriculum structure to follow the essential pillars that Hoffmann and Borduka (2009) and Cibula and Kesic (2009) describe in their papers would be a step forward to adapt a novel concept in the current GO education. This translates in the introduction of several NTS modules that are essential to optimise surgical skills training in each sub-speciality and solidifying the trainer’s perspective as to what would be essential for the GO surgeons to know. The presence of advanced laboratory facilities and adequate funding will also rapid evolution of the course to meet the future needs of GO. As increasing emphasis is given in structured assessment of technical and NTS, a recent systematic review (Hanrahan et al., 2021) can act as a guide for a formal assessment framework for the course and standardise its educational value in the GO community.

An ambitious idea would be to adapt novel curricular architect concepts like the “iG4” one (Sideris et al., 2015; Sideris et al., 2020; Theodoulou et al., 2020), where technical skills education is basically merged in a structured manner with several NTS modules, along with applied surgical science workshops and basic science principles to provide holistic education. “Omnigon iG4” is an adaptable curriculum concept (Sideris et al., 2021) which employs the principle of holistic surgical education and could essentially re-shape the curriculum of an advanced cadaveric course to meet the “holistic” needs of the future surgeon, delivered in a standardised fashion.

## Conclusions

This course is an excellent concept to advance GO surgical based education and clearly serves the future idea of multi-specialty, expert-based involvement in advanced cytoreductive surgery. “Learning from the experts” seems to be a promising concept to meet the rapidly increasingly demand for excellence in GO surgery. Adapting the course curriculum to a more standardised fashion to meet the general principles of most UK, US or European training curricula will solidify the effort to achieve excellence in the learning outcomes and make the course an essential adjunct of most GO fellowships. Although COVID 19 has significantly changed the status and delivery forms of SBL education, it is almost certain that such advanced postgraduate course will continue to evolve in a “face-to-face” delivery mode, employing several innovative adjuncts to optimise the educational value and benefits. Ultimately, more research should be designed and focused to identify the impact of such courses directly on patients’ outcomes.

## Appendix I.

**Evaluation approach & synthesis.**

Questions
**Q1-21**
Q1 Years of consultant experience
Q2 Practice in the UK (Y/N):
Q3 Place of practice (Region):
Q4 This course’s objectives are essential for GO training (scale 0-5):
Q5 This course met pre-set objectives (scale 0-5):
Q6 This course should be part of the Gynae onc sub-spec curriculum (scale 0-5):
Q7 On a scale 0-5 how confident are you in performing cytoreductive surgery related to HPB:…, Colorectal:…., Urology:…., Upper GI:…..
Q8 On a scale 0-5 which part of the course was more useful: HPB:…, Colorectal:…., Urology:…., Upper GI:…..
Q9 Which parts of the course (HPB, Colorectal, Urology, Upper GI) would you wish more exposure on during the course?
Q10 Theoretical lectures were useful (scale 0-5)…
Q11 Theoretical lecture should be less next time (Y/N/about right amount):……..
Q12 Cadaveric simulation was the ultimate simulation modality for this course:……….
Q13 This course made me more confident in HPB related cytoreductive procedures (scale 0-5)
Q14 This course made me more confident in Colorectal related cytoreductive procedures (scale 0-5)
Q15 This course made me more confident in Upper GI related cytoreductive procedures (scale 0-5)
Q16 This course made me more confident in Urology related cytoreductive procedures (scale 0-5)
Q17 I need to re-attend this course to consolidate my knowledge on HPB:…, Colorectal:…., Urology:…., Upper GI….. (scale 0-5)
Q18 I would recommend this course to a Sub-specialty Trainee (scale 0-5):….
Q19 I would recommend this course to a junior consultant (scale 0-5):…..
Q20 I would recommend this course to a senior consultant (scale 0-5):……
Q21 Facilities were adequate for this course (scale 0-5):
Introduction and instrumentation scale Day 1 (0-5):…..
Introduction and instrumentation scale Day 2 (0-5):…..
**D1 HPB cadaveric modules:**
-exposure (scale 0-5):..
-performance improvement (scale 0-5):…
-applicability in my practice (scale 0-5):…
-anatomy of the right upper abdomen (scale 0-5):….
**D2 Upper GI modules:**
-exposure (scale 0-5):..
-performance improvement (scale 0-5):…
-applicability in my practice (scale 0-5):…
-anatomy of the left upper abdomen (scale 0-5):….
**D3 Urology modules:**
-exposure (scale 0-5):..
-performance improvement (scale 0-5):…
-applicability in my practice (scale 0-5):…
-anatomy of the urinary tract (scale 0-5):….
**D4 Colorectal modules:**
-exposure (scale 0-5):..
-performance improvement (scale 0-5):…
-applicability in my practice (scale 0-5):…
-anatomy of the bowel (scale 0-5):….
**Open space feedback**
Areas to improve:
Areas to include next time:
Areas that should be expanded more:
Facilities:
Any other comment/feedback:
